# Exploring the dynamics of reactive oxygen species from CaviPlasma and their disinfection and degradation potential — the case of cyanobacteria and cyanotoxins

**DOI:** 10.1007/s11356-024-35803-4

**Published:** 2024-12-20

**Authors:** Klára Odehnalová, Jan Čech, Eliška Maršálková, Pavel Sťahel, Barbora Mayer, Vinicius Tadeu Santana, Pavel Rudolf, Blahoslav Maršálek

**Affiliations:** 1 Department of Experimental Phycology and Ecotoxicology, Institute of Botany, Czech Academy of Sciences, Lidická 25/27, 602 00 Brno, Czech Republic; 2 Department of Plasma Physics and Technology, Faculty of Science, Masaryk University, Kotlářská 267/2, 611 37 Brno, Czech Republic; 3 Central European Institute of Technology, Brno University of Technology, Purkyňova123, 612 00 Brno, Czech Republic; 4 V. Kaplan Department, Faculty of Mechanical Engineering, Brno University of Technology, Technická, 2896/2, 616 69 Brno, Czech Republic

**Keywords:** Plasma-treated liquid, Electric discharge, Hydrodynamic cavitation, Radicals, Water treatment, Cyanobacteria, Microcystins

## Abstract

We investigated the production of highly reactive oxygen species (ROS) in solutions undergoing treatment using CaviPlasma (CP) technology. This technology combines plasma discharge with hydrodynamic cavitation. This study focused on factors such as pH, conductivity, presence of salts and organic matter affecting ROS formation and their stability in solutions. Depending on the used matrix, CP produces 450–580 µg L^−1^ s^−1^ of hydrogen peroxide and 1.9 µg L^−1^ s^−1^ of hydroxyl radicals dissolved in liquid. Using cyanobacteria and cyanotoxins as example, we proved that CP technology is a highly efficient method for destroying microorganisms and persistent toxins. The biocidal effect of the CP treatment was confirmed on two species of cyanobacteria, *Synechococcus elongatus* and *Merismopedia minutissima*. The effectiveness of the technology in degrading microcystins was also demonstrated. The potential of this technology is based on its high energy efficiency, G(H_2_O_2_) ≈ 10 g kWh^−1^ and G(O_3_) ≈ 0.03 g kWh^−1^ (in deionised water), realistic applicability with throughput rates (> 1 m^3^ h^−1^), and comparatively easy scalability system.

**Supplementary Information:**

The online version contains supplementary material available at 10.1007/s11356-024-35803-4.

## Introduction

Currently, extensive research is being conducted on using non-thermal plasma (cold plasma) for biological decontamination. Plasma processes are generally regarded as a combined process of advanced oxidation processes (AOPs), including ozonation, UV photolysis, pyrolysis, etc. While the exact mechanism for bacterial deactivation is still being studied, two main theories have been suggested: electrostatic disruption of cell membranes (electroporation) and lethal oxidation of membrane or cytoplasmic components. Plasma treatment has been found to be highly effective in inactivating bacteria, as demonstrated by various studies (Gaunt et al. 2006; Laroussi 2005; Tendero et al. 2006). The interaction between non-equilibrium plasma and liquid is of fundamental importance in various applications such as materials science, environmental remediation and healthcare (Bruggeman et al. 2016). In cold atmospheric-pressure plasma, high densities of reactive species are generated from dissociation and recombination processes. The plasma-initiated ultraviolet (UV) photolysis generates OH species, resulting in hydroxyl radicals (·OH) becoming a major reactive oxygen species (ROS) in the solution (Attri et al. 2015; Hu et al. 2022). Alongside hydroxyl radicals, other short-lived species such as superoxide (^1^O_2_), superoxide anion radicals (·O_2_^−^), hydroperoxyl radical (·OOH), nitric oxide and dioxide radical (·NO, ·N_2_O), peroxynitrite (ONOOH, OONOOH), and a long-lived species such as H_2_O_2_, ozone, molecular hydrogen, and oxygen are formed (Bruggeman and Leys 2009, Hu et al. 2022). Generally, the half-life of short-lived species lasts microseconds to seconds, while that of long-lived ROS is days to months. The efficiency of gas–liquid processes depends on design and operating parameters, types of contact operations and fluid properties. In fundamental mass transfer theories, increasing the contact area is essential to improve process performance.

Cyanobacteria are essential photoautotrophic microorganisms that have a significant role in providing oxygen and maintaining ecological balance. However, they are becoming overabundant due to excessive human nutrient inputs and climate changes. As a result, cyanobacterial blooms producing toxic secondary metabolites, cyanotoxins that can harm the ecosystem and pose a public health risk, are formed onto the surface water bodies. (Metcalf and Codd 2020; Svircev et al. 2017, 2019; Van Goethem and Cowan 2019). Cyanotoxins vary considerably in their chemical nature and structure, toxic potency, mode of action and organs affected. They are considered one of the most lethal groups of biotoxins known. Among them, hepatotoxic microcystins (MC) are identified as monocyclic heptapeptides with a common structural element consisting of 3-amino-9-methoxy-10-phenyl-2,3,8-trimethyldeca-4,6-dienoic acid (Adda), N-methyldehydroalanine (Mdha), D-alanine, β-linked D-erythro-β-methylaspartic acid, and β-linked D-glutamic acid. This group counts more than 100 congeners (Rinehart et al. 1994; Schneider and Bláha 2020). In the natural freshwater bodies infested with cyanobacteria, MC are the most prevalent cyanotoxins, generally in concentrations of sub µg/L to a few hundred µg/L (El Herry et al. 2008; Preece et al. 2017; Yen et al. 2011). Depending on environmental factors, MC production can vary among species and even within the same species. Environmental variables such as light intensity, temperature, pH, and nutrient availability can affect cyanobacterial growth and microcystin production (Codd et al. 2005; Dai et al. 2016; Natumi and Janssen 2020).

Recently, our team developed a newly patented technology combining hydrodynamic cavitation with electric discharge — CaviPlasma (Cech et al. 2020; Rudolf et al. 2019). In this CaviPlasma (CP) technology, formerly called hydrodynamic cavitation plasma jet (HCPJ), the biocidal liquid is generated using electric discharge initiated in the vapor–liquid environment of a hydrodynamic cavitation cloud (Gogate and Patil 2015). This setup enables the advantageous generation of plasma in the low-pressure gaseous phase of cavities consisting of saturated water vapours. The discharge is ignited in a cavitation cloud formed after passing the liquid through a constriction (Venturi nozzle) (Rudolf et al. 2013). The fine bubbles (cavities) formed downstream the Venturi nozzle throat increase the efficiency of gas–liquid interface processes by increasing the contact surface area, a key factor of process performance (Bruggeman et al. 2016; Temesgen et al. 2017). Reactive species in the plasma-treated liquid could form not only through reactions with the gas-phase constituents formed by the discharge but also through photolysis by plasma-emitted UV photons. The resulting plasma-chemical products (radicals, atoms and molecules) are finally dissolved into the liquid phase (water) after the collapse of hydrodynamic cavitation cloud. This process occurs in the presence of a strong increase in the pressure and temperature of the gas inside cavities during their collapse phase (Sivakumar and Pandit 2002).

The CaviPlasma technology has undergone further development and modifications since the first published results on 1st generation of CaviPlasma (Cech et al. 2020; Marsalek et al. 2020), especially regarding electrode material, discharge regime and stability, and system efficiency. In 1st generation CaviPlasma, the cavitation cloud does not bridge the distance between discharge electrodes, leaving only the nozzle electrode in direct contact with the discharge. In the current 2nd generation CP, the cavitation cloud was prolonged, and both electrodes were exposed to the discharge, resulting in increased efficiency of hydrogen peroxide production. Thus, in this report, we focused on verifying the production of reactive oxygen species produced by 2nd generation CP, their concentration and efficiency in removing cyanobacteria as well as their toxins. The species *Synechococcus elongatus* and *Merismopedia minutissima* were chosen for this study because they are suitable for studying using the picocyanobacteria and colonial cyanobacteria models.

## Material and methods

### Materials

Titanium (IV) oxysulfate solution (Ti, ~ 5%; H_2_SO_4_, 27–31%), terephthalic acid (98%), 2-hydroxy terephthalic acid (97%), 5,5-dimethyl-1-pyrroline N-oxide (DMPO; ≥ 98.0%) and 2,2,6,6-tetramethyl-4-piperidinol (TMP-OH; 98%) were purchased from Merck Life Science s.r.o. (Prague, Czech Republic). Hydrogen peroxide solution (30%) and sodium salts (chloride, sulphate, carbonate and nitrate) were purchased from PENTA s.r.o. (Prague, Czech Republic). Methanol, acetonitrile and formic acid (for LC–MS) were from VWR International (Stříbrná Skalice, Czech Republic).

Certified MC standards (MC-RR, MC-YR, MC-LR, MC-WR, MC-LA, MC-LW, MC-LY, MC-LF;) were purchased from Alexis Biochemicals (Läufelfingen, Switzerland).

### CaviPlasma technology

**Fig. 1:**
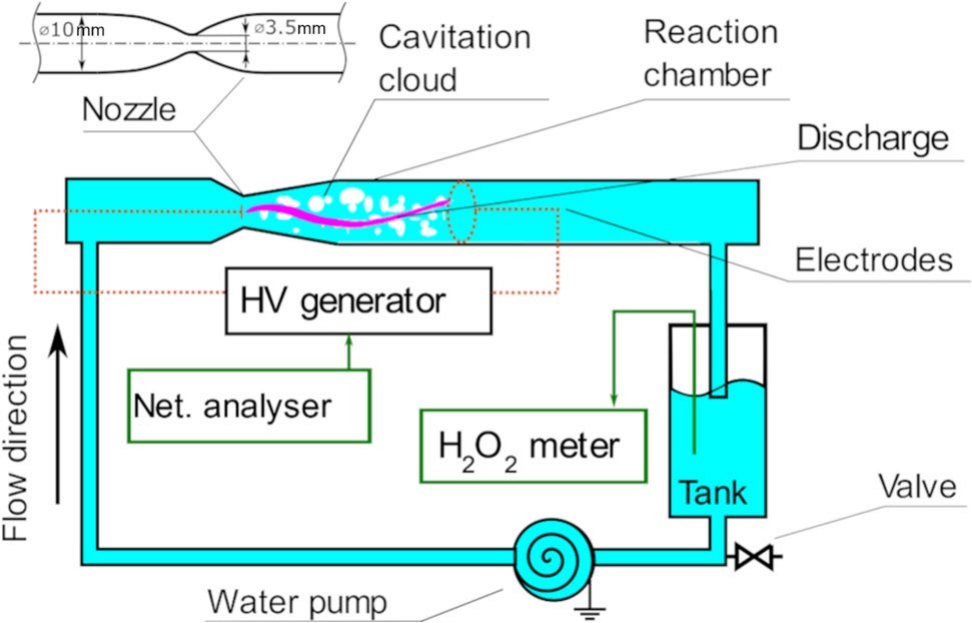
The scheme of the plasma treater unit utilising CaviPlasma technology

The CaviPlasma unit consists of a hydraulic circuit producing a hydrodynamic cavitation environment in fast-flowing liquid in the reaction chamber, the high-voltage (HV) subsystem sustaining electric discharge in the cavitation cloud and retention/recirculation tank for storage of plasma-treated liquid. The scheme of the plasma apparatus is given in Fig. 1. The treatment conditions were followed using a network analyser (measuring the input electric power), flowmeter and hydrogen peroxide sampling and analysis.

The experiments were performed in batch mode of operation under the following conditions. The flow rate through the cavitation chamber was set to 1.3 m^3^ h^−1^ using Calpeda MXHM 205/A pump and monitored using Keyence FD-H32 flowmeter. Venturi tube manufactured from polycarbonate tube (10 mm inner diameter) has a smooth shape of converging–diverging nozzle created by B-splines with constriction of 3.5 mm in diameter. Cavitation conditions of CaviPlasma unit operating point are characterised by cavitation number, upstream velocity and temperature of the liquid (Sarc et al. 2017). The cavitation number is defined based on downstream pressure (*p*_*2*_), throat velocity (*v*_*throat*_*)*, liquid density (*ρ*) and vapour pressure (*p*_*vapor*_) (Franc and J.-M 2010).

1 $$\sigma =\frac{{p}_{2}-{p}_{vapor}}{\rho \frac{{v}_{throat}^{2}}{2}}=0.65$$

The velocity upstream of the throat is 4.6 m s^−1^. Vapor pressure was computed according to IAPWS (Wagner and Pruss 2002) as a function of the temperature.

Liquid solution treated by CaviPlasma was based on deionized water, detailed composition for the individual test is provided below. It should be noted that the liquid solution was not deaerated prior to the experiment, i.e. gas content corresponded to partial pressure under normal conditions (room temperature, atmospheric pressure) as stated by Henry’s law.

In the reaction chamber made of a polycarbonate tube of an inner diameter of 10 mm with the Venturi nozzle constriction (throat diameter 3.5 mm), the fast-flowing liquid produces approx. 14-cm long hydrodynamic cavitation cloud (HCC) in which the electric discharge was sustained between a titanium electrode inserted in the nozzle and a ring-shaped steel electrode (chrome-plated) 12-cm apart from the nozzle electrode downstream. The plasma channel was ignited using the alternating high voltage at a frequency of approx. 33 kHz, HV peak-to-peak amplitude value of approx. 10 kV and peak current of approx. 1 A. The power input to the plasma generator was set to 1 kW and monitored using an Orbit Merret OM 402PWR network analyser. As an indicative measure, the concentration of H_2_O_2_ in the resulting plasma-treated liquid was measured using a Quantofix Relax tester with MACHEREY–NAGEL QUANTOFIX Peroxide 100 test strips (MACHEREY–NAGEL GmbH & Co. KG, Dueren, Germany).

The treatment dose was set using so-called specific input energy (SIE), which represents the amount of energy delivered to the one litre of treated liquid using the HV generator. Different plasma-treated media (PTM) were prepared and tested using three plasma treatment doses, corresponding to 8.4 kJ L^−1^, 25.2 kJ L^−1^, and 42 kJ L^−1^, which is equivalent to the running times of respectively 42 s, 126 s, and 210 s of the plasma treatment of 5 L batches. The effective values of the total residence time of PTM in the reaction zone were estimated to be approx. 0.08 s, 0.23 s and 0.39 s, respectively, being the upper estimation supposing the uniform flow through the reaction chamber after HCC collapse.^1^ The pH, conductivity, and temperature of the treated solutions were monitored using Hanna Combo pH/EC/TDS/Temp tester (Hanna Instruments Czech s.r.o., Praha, Czech Republic) after each treatment.

### ROS measurements

The quantity of H_2_O_2_ was estimated by a colorimetric method based on a specific reaction between hydrogen peroxide and titanyl ions (Eisenberg 1943). The reaction produces a yellow pertitanic acid complex with an absorption peak maximum at 407 nm. For the quantification, a portion of 1 mL of the sample was mixed with titanyl reagent (0.5 mL) and the absorbance was measured using a SparkTM spectrophotometer (Tecan, Austria).

The ozone content was determined spectrophotometrically using a commercially available cuvette test from Hach Lange (LCK310; range 0.05–2.0 mg L^−1^ O_3_), based on the ozone reaction with diethyl-p-phenylenediamine to form a red dye. Water samples treated with CaviPlasma were collected immediately after treatment and mixed with the cuvette test reagents. The product's absorbance was measured on a Hach Lange DR2800. The measured values were corrected to the presence of hydrogen peroxide.

For nitrite monitoring in all matrices, the commercially available cuvette test from Hach Lange (LCK541; range 0.005–0.1 mg L^−1^ NO_2_^−^), based on the diazotisation reaction, forming intensively coloured azo dye was used.

Free radicals are highly reactive molecular species with a short half-life. Therefore, the spin trapping technique based on the formation of long-lived and EPR-detectable spin adducts was used for their detection. The measurements were performed in a flat quartz cuvette on an X-band EPR spectrometer (9.5 GHz) assembled with a JEOL JM-PE-3 resistive magnet and a Magnettech MXH2 microwave source and control unit. A magnetic field modulation amplitude of 0.5 G at 100 kHz and 20 mW of microwave power was used. Depending on the experiment, spectra were accumulated and averaged to improve the signal-to-noise (SNR) ratio. In our experiments, we used two types of spin traps: 2,2,6,6-tetramethyl-4-piperidinol (4-hydroxy-TEMP) as a specific spin probe to detect singlet oxygen and 5,5-dimethyl-1-pyrroline-N-oxide, which is commonly applied to detect hydroxyl (·OH), or hydroperoxyl (·HO_2_) and superoxide anion (·O_2_^−^) radicals. Immediately after the treatment, a portion of 90 µL of the sample was added to 10 µL of a spin trap (c_DMPO_ = 2.5 M or 5 M; c_TMP-OH_ = 0.25 M), and the mixture was subsequently frozen in liquid nitrogen and thawed before being measured. In the presence of singlet oxygen, the EPR silent species 4-hydroxy-TEMP becomes the stable radical 4-hydroxy-TEMPO, which is EPR active (Lion et al. 1980). The increased area of the 4-hydroxy-TEMPO signal obtained by fitting the EPR spectra with Lorentzian line shapes is directly proportional to the presence of singlet oxygen in the samples. The EPR spectra of the DMPO adducts were simulated using Easyspin (version 6.0.0-dev54) (Stoll and Schweiger 2006), a package for EPR spectral simulation working under MATLAB (The Mathworks, Inc., Natick, MA 01760, 2023). The DMPO spectra contained several components that were identified according to the hyperfine coupling constants, and the relative concentration of radicals was based on the area of each component among different samples.

For ·OH quantification, a reaction with terephthalate anions (2 mM) was used (Mason et al. 1994). The fluorescence was measured using a SparkTM spectrophotometer (Tecan, Austria).

### Cyanobacterial culture

Cyanobacteria *Synechococcus elongatus* KOVROV 1972/8 and *Merismopedia minutissima* (Strain CEPÁK 1994/1) stock cultures that were obtained from the Culture Collection of Autotrophic Organisms (CCALA) (Třeboň, Czech Republic) were chosen as test organisms. Cyanobacterial cultures were grown in sterilised ZBB media (see detail in S7) prepared from a 1:1 mixture of Z-medium (Zehnder and Staub medium) and BB-medium (Bristol and Bold medium) in 100 mL Erlenmayer flasks at 24 ± 1 °C under continuous illumination (90 μmol m^2^ s^−1^) by fluorescent lamps (Phillips, TLD 36 W/33). The media were sterilised by autoclaving using warm, moist air at elevated pressure. This process used a steam temperature of 121 °C for a period of 23 min and a pressure of 101.3 kPa. The growth of the cultures was monitored by measuring the optical density at 665 nm (OD). The cyanobacterial biomass was collected from the cultures approaching the stationary phase of the growth. Under the experimental conditions, the cultures were found to be in the middle of the exponential growth phase within a period of 10 to 12 days.

Samples containing cyanobacteria were filtered through a nylon syringe filter (0.45 µm, nylon) before further chemical analysis.

### Quantification of chlorophyll and photosynthetic activity

Chlorophyll content was determined using the bbe Moldaenke FluoroProbe (Schwentinental, Germany), which allows the selective quantification of phytoplankton groups (cyanobacteria, green algae, diatoms and cryptophytes) and their amount is expressed in µg L^−1^ Chlorophyll *a* (Gregor et al. 2007).

To determine the photosynthetic activity in cyanobacterial cell suspensions, a pulse amplitude modulated (PAM) technique with AquaPen AP110-C (Photon System Instruments, Drásov, Czech Republic) was performed. This technique derives the activity data from the chlorophyll fluorescence kinetics.

### Microcystins extraction and quantification

Microcystins were extracted from wild-type cyanobacteria collected in July 2018 from the Brno Reservoir. After collection, the cells were lyophilised and kept in a dry, dark place. For MC extraction, 30 ml of 50% methanol was added to a total of 0.6 g of cell debris, and the mixture was vortexed for 3 min at 3000 rpm, followed by 5 min in an ultrasonic bath. The procedure was iterated three times. The extract was centrifuged (4000 × g for 15 min), and the supernatant was filtered through a nylon syringe filter (pore size 0.45 µm). The quantification of MC was accomplished by LC–MS/MS procedure.

MC were quantified using an Agilent 1260 Infinity high-performance liquid chromatography system (Agilent Technologies, CA, USA) coupled to an Agilent 6460 TripleQuad mass spectrometer (Agilent Technologies, CA, USA) equipped with an electrospray ionisation (ESI) interface. The details are given in Supplementary information (page S3).

For microcystin degradation experiments, the deionised water was fortified with MC to give a final microcystin concentration of 3–5 µg L^−1^. The solution was directly treated in CP unit as stated above, and the microcystin content was quantified by LC–MS/MS after solid phase extraction (more information on the procedure is given on pages S3 and S4 in the Supplementary information).

### Data evaluation

The data were processed using standard Microsoft Excel tools. If not stated otherwise, each experiment was replicated three times, and the data were presented as mean ± standard deviation.

## Results and discussion

### Analysis of short-lived species

Since free radicals, containing one or more unpaired electrons, are generally short-lived, they cannot (in most cases) be observed directly in the liquid solution. Therefore, spin trapping of the radicals and subsequent electron paramagnetic resonance (EPR) analysis of the more stable adducts is performed. As spin traps, organic reagents such as nitrones or chelated metal complexes, allowing the detection of ·OH, ·OOH, ·NO and carbon-centred radicals (Gorbanev et al. 2016; Halliwell and Gutteridge 2015; Hu et al. 2022).

**Fig. 2:**
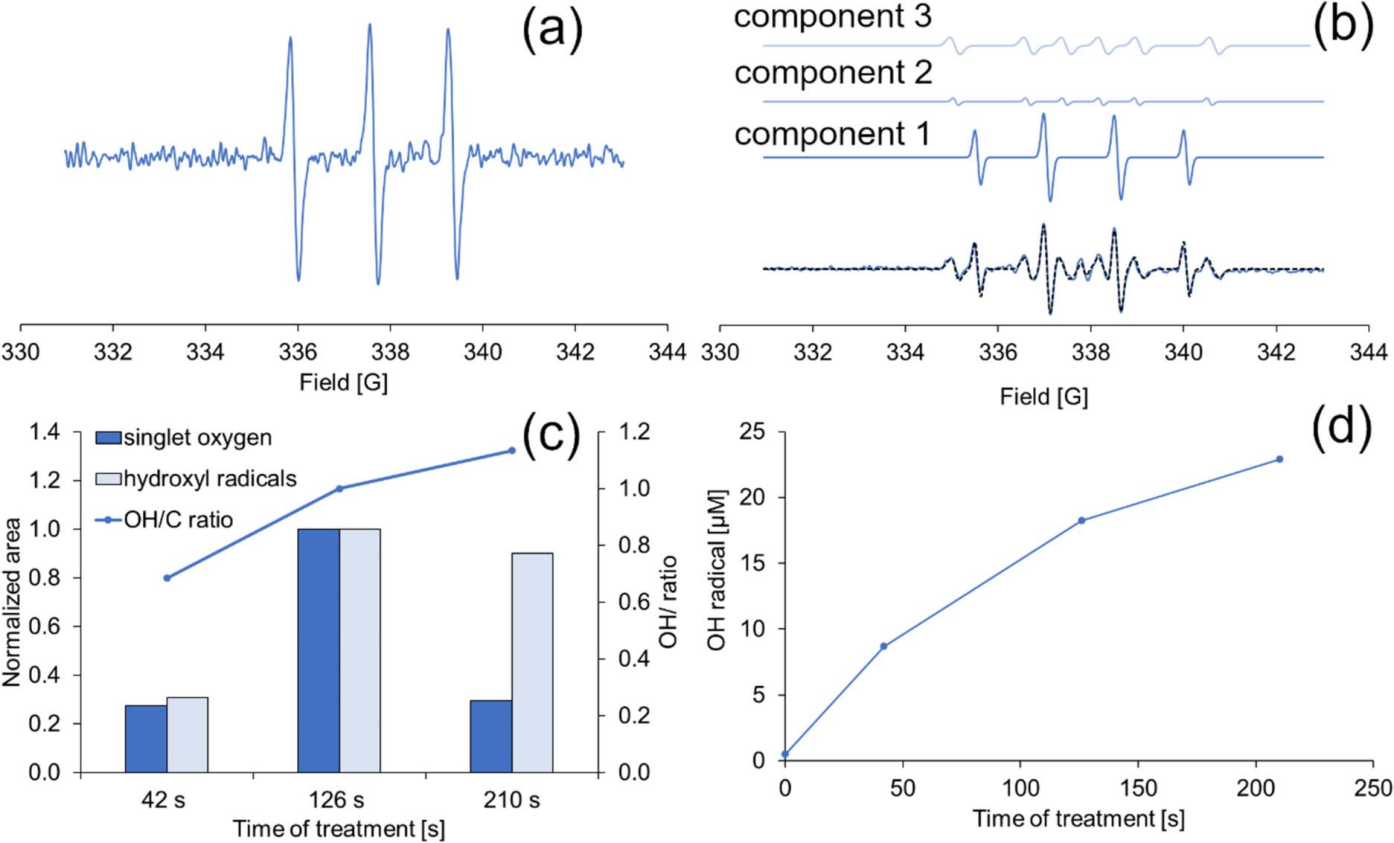
Typical experimentally obtained spectra of singlet oxygen adduct with 4-hydroxy-TEMP (**a**), obtained (blue line) and simulated (dashed black line) spectra of DMPO adducts (**b**), formation of radicals after different CaviPlasma treatment doses (**c**), and relation of ·OH concentration to treatment time (**d**). Experiments were performed in deionised water. Component 1 represents the simulated ·OH spectrum, while components 2 and 3 belong to carbon-based radicals

The formation of singlet oxygen under different CaviPlasma treatment doses using 4-hydroxy-TEMP spin trap was monitored. Figure 2a depicts the typical experimentally obtained spectrum of the 4-hydroxy-TEMP adduct with ^1^O_2_. To identify the ·OH, a DMPO spin trap was used, and the experimentally obtained and simulated DMPO-OH spectra are presented in Fig. 1b, with hyperfine coupling constant a_N_ = 15.2 ± 0.3 G and a_H_ = 14.7 ± 0.3 G. In addition to DMPO-OH adduct (Fig. 2b), signals of a carbon-centred radical were also detected. Though the structure of the adducts was not precisely determined, the simulated hyperfine values (a_N_ = 15.5 ± 0.3 G and a_H_ = 22.9 ± 0.4 G; a_N_ = 15.9 ± 0.1 G and a_H_ = 23.5 ± 0.2 G) matched the literature values for various carbon-centred radical adducts of DMPO (Buettner 1987). The carbon-centred adducts could have been formed as a result of DMPO degradation in the presence of other ROS (Gorbanev et al. 2016). This claim was confirmed when 4-hydroxy-TEMP was added to trap singlet oxygen, which significantly reduced the carbon-centred adduct signals.

As shown in Fig. 2c, the density of formed radicals increased between the 42-s and 126-s treatments. However, as the treatment time was extended to 210 s, the concentration of singlet oxygen and hydroxyl radical decreased. Although the hydroxyl radical content decreased slightly, a significant decrease was noticed in the case of singlet oxygen. In contrast, the ratio of ·OH to carbon-centred radicals increased. The decrease in radical production can most likely be explained by their mutual recombination and formation of other ROS (not limited to radicals only; see below). The slowing trend of hydroxyl radical formation with continued treatment time is shown in Fig. 2d when it was monitored using terephthalic acid.

**Fig. 3:**
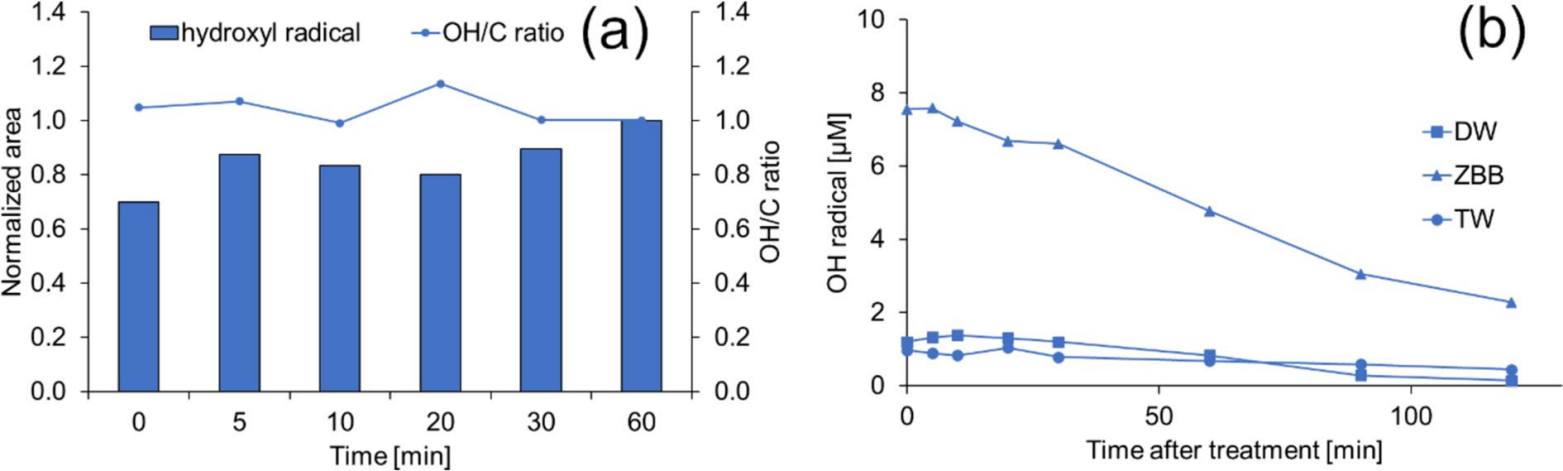
Concentrations of hydroxyl radicals measured in deionised water via spin trapping (**a**) and terephthalic ions in different matrices (**b**) as a function of post-treatment time. DW, ZBB and TW denote deionised water, culture medium and tap water, respectively

Once in the liquid phase, the lifetimes of plasma-generated species are determined by their reactivity and the local concentration of reaction partners. If there are organic compounds present in the liquid, the reaction of ·OH with these compounds can sustain reactivity by forming new radicals. This can trigger further reactions. Generally, ·OH reacts rapidly and unselectively with ionic species and biologically relevant molecules, with high rate constants ranging from 10^7^ to 10^10^ M^−1^ s^−1^ (Halliwell and Gutteridge 2015). For example, when two ·OH meet, they form H_2_O_2_ at a rate constant of 5 × 10^9^ M^−1^ s^−1^, while the rate constant for reaction with H_2_O_2_ is lower being 2.7 × 10^7^ M^−1^ s^−1^. Although these reactions take place during plasma discharge (Bruggeman et al. 2016; Gorbanev et al. 2016), they would not need to occur in deionised water because the steady-state ·OH concentration is small. Attri et al. (2015) observed that the lifespan of hydroxyl radicals in deionised water increases with increasing solution depth during plasma-initiated photolysis. Their results suggest that interaction with water molecules reduces the rate of other reactions. Their theoretical calculations supported these claims. Thus, although the radicals are considered to be short-lived, their lifespan was tested in the time range of 0 to 60 min. This experiment is essential in terms of the use of so-called plasma-activated water (PAW). In this experiment, deionised water was treated for 210 s in a CP unit and then sampled into vials containing DMPO after standing for 5, 10, 20, 30 and 60 min in transparent sealed glass containers (21 °C, common room light condition). The experiment's results are displayed in Fig. 3a. This figure indicates that the hydroxyl and carbon radical content and their ratio remained constant. Again, the EPR radical determination results were verified by reactions with terephthalic ions (in all three studied matrices for up to 2 h and 210 s CP modification) and are shown in Fig. 3b.

As suggested earlier (Attri et al. 2015), the radical’s increased lifetime could be due to the hydrogen bonding with water molecules, which subsequently reduces the reaction rate with surrounding molecules. It is important to note that, even in the aforementioned study, the observed lifespan was still in the order of microseconds. However, in our case, the pump continuously drove the solution, significantly accelerating the radical diffusion in the aqueous environment compared to the movement by mere diffusion in the aforementioned study. The formation of hydrogen bonds in the gas phase has been described by Dubey et al. (1997). According to their research, the probability of entropic restriction of reactions between hydroxyl radicals and water increases due to the formation of a complex between ·OH and the water molecule. This complex increases the chance of entropic limitation of the reaction. Along with radicals, hydrogen peroxide and ozone are also formed during the treatment (see Section 3.2). Thus, a possible explanation of “prolonged” ·OH presence in the solution could result from continuous addition via the peroxone process, even after an hour of still standing. In the peroxone process, H_2_O_2_ initiates the decomposition of O_3_ to form radical forms such as ·OH (Asghar et al. 2022; Staehelin and Hoigne 1982). The molar ratio of H_2_O_2_/O_3_ corresponding to the stoichiometric decomposition of O_3_ is 0.5. The rate of radical formation or extinction of ozone is determined by its rate constant (k = 2.8 × 10^6^ M^−1^ s^−1^). Additionally, the gradual release of hydroxyl radicals during the collapse of nanobubbles generated by hydrodynamic cavitation presents another potential source. Nanobubbles have unique properties due to their small size, including longevity, high gas solubility, and enhanced mass transfer. These nanobubbles could persist in solution for extended periods, as observed by Soyluoglu et al. (2021), (2023). If present, they could contribute to the presence of hydroxyl radicals persisting beyond the treatment period, either through the energy release, causing water molecule cleavage during their collapse or by the reaction of the released ozone trapped in the cavities. Therefore, in the next phase of the experiments, the processes contributing to radical longevity should be carefully investigated to verify their contribution to the observed phenomenon.

### Analysis of long-lived species

Hydrogen peroxide and ozone are formed as non-radical ROS during the plasma treatment of water. Several possible reactions can lead to their formation during plasma-chemical reactions (Hu et al. 2022; Jiang et al. 2014; Locke and Shih 2011). In the gas phase, the primary production reaction for H_2_O_2_ is typically the three-body reaction of two ·OH and a collision partner, which can be any atom or molecule (Gorbanev et al. 2018; Vasko et al. 2014). Therefore, the rate of H_2_O_2_ formation is sometimes used as a relative indicator of hydroxyl radical formation. When formed in the gas phase (according to Gorbanev et al. 2018), they dissolve into water according to Henry’s law solubility constants. Ozone is less stable than hydrogen peroxide and decomposes in neutral and basic solutions by a cyclic chain mechanism to form hydroxyl radicals (Glaze 1987). Additional radicals could be created in the presence of peroxide and ozone during plasma-chemical reactions (Staehelin and Hoigne 1982). Thus, the qualitative and quantitative analysis of these oxidants is essential. However, their content depends on multiple factors, mainly the pH of the solution and the presence of salts, etc. Therefore, we evaluated the formation of these ROS in pure and deionised water fortified with chloride, nitrate, sulphate and carbonate (as sodium salts) to an anion concentration of 10 mg L^−1^.

The CaviPlasma unit's treatment process yields hydrogen peroxide proportional to the treatment time. The CP unit produces approximately 450—580 µg L^−1^ s^−1^ of hydrogen peroxide, depending on the matrix used (Table 1 and Fig. S1a). It’s worth noting that hydrogen peroxide production outpaces hydroxyl radical production by more than 100-fold (R = 0.98; Fig. S1c). However, it’s crucial to note that ·OH are detected in the liquid phase, while the main plasma-chemical reactions occur in the gas phase. Hence, the significant ratio of H_2_O_2_ to ·OH aligns well with Gorbanev’s findings, suggesting the potential formation of H_2_O_2_ in the gas phase. At the same time, we cannot dismiss the possibility of other reactions besides hydroxyl radicals, being the source of hydrogen peroxide. In deionised water, the ozone concentration formed after 210-s CP treatment was approximately 270 times lower than the hydrogen peroxide content. In comparison to deionized water, ozone production in the presence of salts was noticeable after a longer treatment duration, as depicted in Fig. S1b. Ozone generation was observed in the presence of sulphates and nitrates after 126 s, and even after 210 s for chlorides and carbonates.

**Table 1:** The hydrogen peroxide content's correlation with the treatment time (dose) in different matrices

Matrix	pH	Conductivity [µS cm^−1^]	Slope [µg L^−1^ s^−1^]	Regression coefficient
Deionised water	6.4	3	581.2	0.9996
NaCl	5.6	21	511.9	0.9999
NaNO_3_	5.7	11	511.3	1.0000
Na_2_SO_4_	6.3	17	512.6	0.9995
Na_2_CO_3_	10.0	22	448.3	0.9998

The highest peroxide and ozone yields were observed for deionised water. Significantly lower yields of H_2_O_2_ and no ozone up to 210-s treatment were produced in the presence of carbonates. As described, carbonate ions act as a scavenger of ·OH and an inhibitor in the cycle of ozone oxidation (Elovitz et al. 2000). Similarly, the chloride anion is also known as an ·OH quencher and an initiator of ozone decomposition. Although Cl^−^ can react with ·OH, the reaction occurs quickly in both directions. Therefore, the impact of Cl^−^ on the quenching of ·OH is usually insignificant. While negligible values of ozone concentrations have been observed in the presence of chloride, its effect on the hydrogen peroxide content was comparable to that of nitrate and sulphate.

Since hydrodynamic cavitation itself produces hydroxyl radicals and thus hydrogen peroxide (Zheng et al. 2022), the contribution of the system setup with and without plasma discharge to these species was also evaluated. When only Venturi nozzle, i.e. pure hydrodynamic cavitation, was engaged, the peroxide content was independent of the treatment time and reached values of 0.7 mg L^−1^ (see Fig. S2). No ozone was detected at the same time. Similarly, while ·OH was detected in both setups, singlet oxygen was not detected when only the Venturi nozzle was employed. Although the trend of ·OH yield was similar (with maxima after 126-s treatment), it was higher for the CaviPlasma unit (see Fig. S3).

**Fig. 4:**
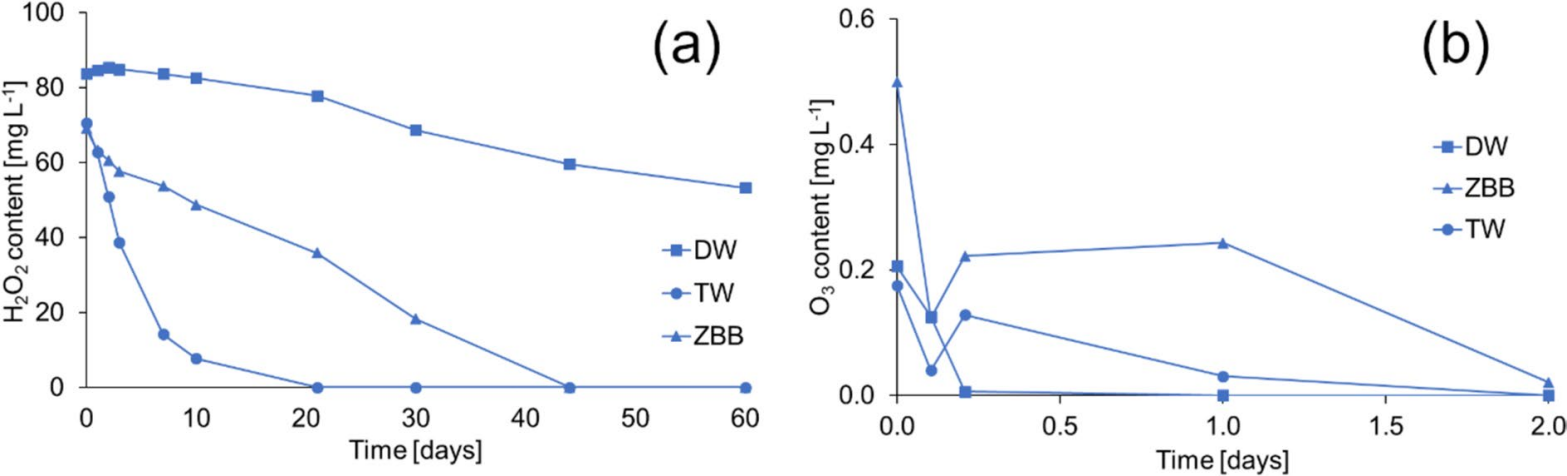
The change of aqueous concentration of hydrogen peroxide content (**a**) and ozone content (**b**) in different matrices after a 210-s treatment. DW, TW and ZBB denote deionised water, tap water and ZBB medium, respectively. The solutions were kept in sealed clear glass bottles under daily light conditions at 21 °C

Furthermore, the hydrogen peroxide and ozone content were monitored after 210-s CaviPlasma treatment (Fig. 4) in three matrices: deionised water (σ = 1.0 µS cm^−1^, pH = 6.5), tap water (σ = 503 µS cm^−1^, pH = 7.6) and ZBB medium (σ = 93.1 µS cm^−1^, pH = 7.0) used for cyanobacterial culture. The composition of ZBB medium is available in Supplementary information on page S7. After the mentioned treatment time, the initial hydrogen peroxide content was highest in deionised water (Fig. 4a). Meanwhile, in tap water, which represents the high conductivity solution, and in ZBB media, as a low conductivity solution, the content was lower but similar. H_2_O_2_ breaks down into water and oxygen through redox reactions that chemical or biological processes can cause. The rate of decay depends on the presence of redox-sensitive metals such as iron or manganese, as well as biological activity. Typically, the decay rates range from a few hours to a few days (Matthijs et al. 2012). Therefore, we followed the content of hydrogen peroxide formed by the CP unit in these matrices for up to 60 days (in closed clear vials at laboratory temperature and common daily light conditions). As can be seen from Fig. 4a, the stability of the hydrogen peroxide content was highest in deionised water, where the content decreased to 64% of the initial concentration after 60 days.

The fastest decrease in hydrogen peroxide content was in tap water, reaching a zero value after 21 days. In the medium sterilised before CP treatment, the hydrogen peroxide content dropped to zero only after visible contamination by microorganisms (on the 30th day).

The ozone content in the deionised and tap water, as well as the ZBB medium, was monitored (Fig. 4b). Surprisingly, in ZBB media, the ozone content measured immediately after passing through the CP unit was nearly triple than in deionised water. The concentration of H_2_O_2_ was similar to that of tap water. The ozone concentration in deionised water decreased to zero within a day, whereas it took two days for zero values to appear in culture medium and tap water. In the latter two media, a slight increase in ozone concentration was observed after five hours, possibly due to the recombination of individual ROS or the decomposition of nanobubbles. In the literature, it could be found that ozone has a lifetime of 10–20 min (at 20–25 °C and atmospheric pressure) and then decomposes to oxygen and water after its production due to a very short half-life in the aquatic environment (Khuntia et al. 2012). However, the results clearly show that ozone lasts in analysed media for a significantly longer time. It is possible that the formation of nanobubbles during cavitation could explain the longevity of ozone in water, similar to the longevity of hydroxyl radicals. These nanobubbles, having a high capacity to hold gas, contain gaseous ozone. They allow the ozone gas to continuously diffuse into the water from the gas–water interface, resulting in a longer-term saturation of the water with ozone (Soyluoglu et al. 2023).

### Hydrogen peroxide and ozone content in the presence of cyanobacteria

**Fig. 5:**
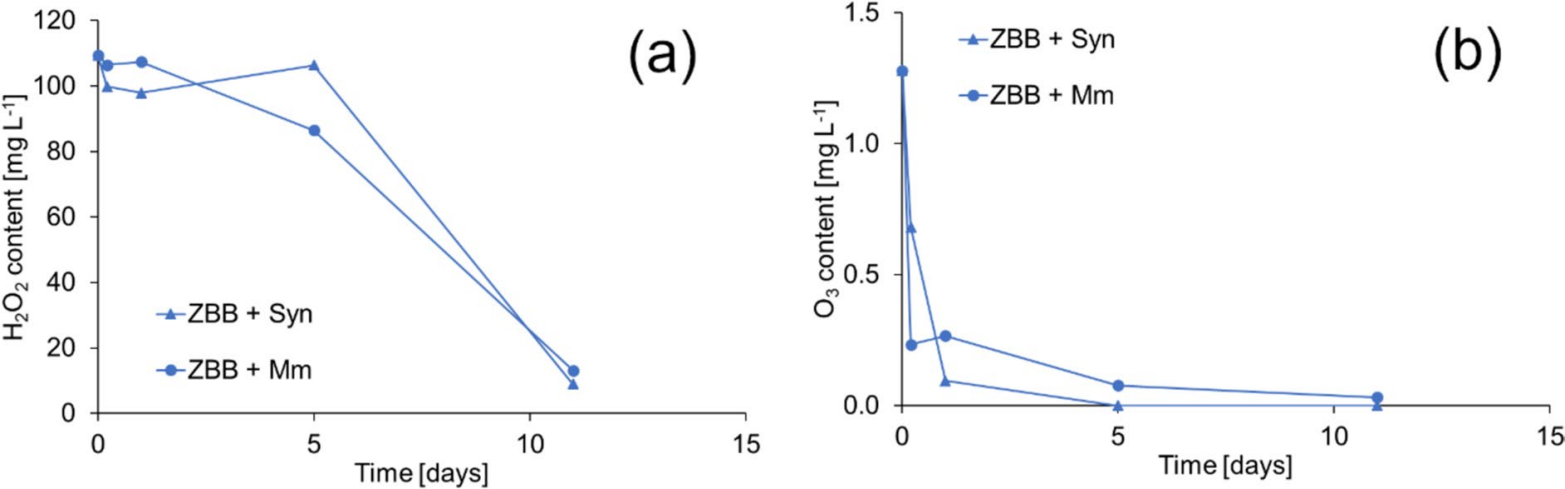
Changes of hydrogen peroxide (**a**) and ozone (**b**) content in treated ZBB medium (PTM) after cyanobacteria inoculation

The formation and behaviour of hydrogen peroxide and ozone were also studied in the presence of cyanobacteria in ZBB medium. The study included two types of experiments that showcased the use of indirect (as PTM) and direct treatments. In the first experiment, the medium was treated with CP, which represented a plasma-treated medium, and then inoculated with cyanobacteria. In this case, the ZBB medium underwent 210-s CP treatment and then was inoculated with cyanobacteria to reach a chlorophyll concentration of 150 ± 25 mg L^−1^. As can be seen in Fig. 5a, a significant decrease (as expected) in hydrogen peroxide content appeared after five days compared to the control CP-treated ZBB medium. While the ozone in the medium alone reached zero within two days (Fig. 4), in the presence of cyanobacteria, we found still measurable values even after several days (Fig. 5b).

**Fig. 6:**
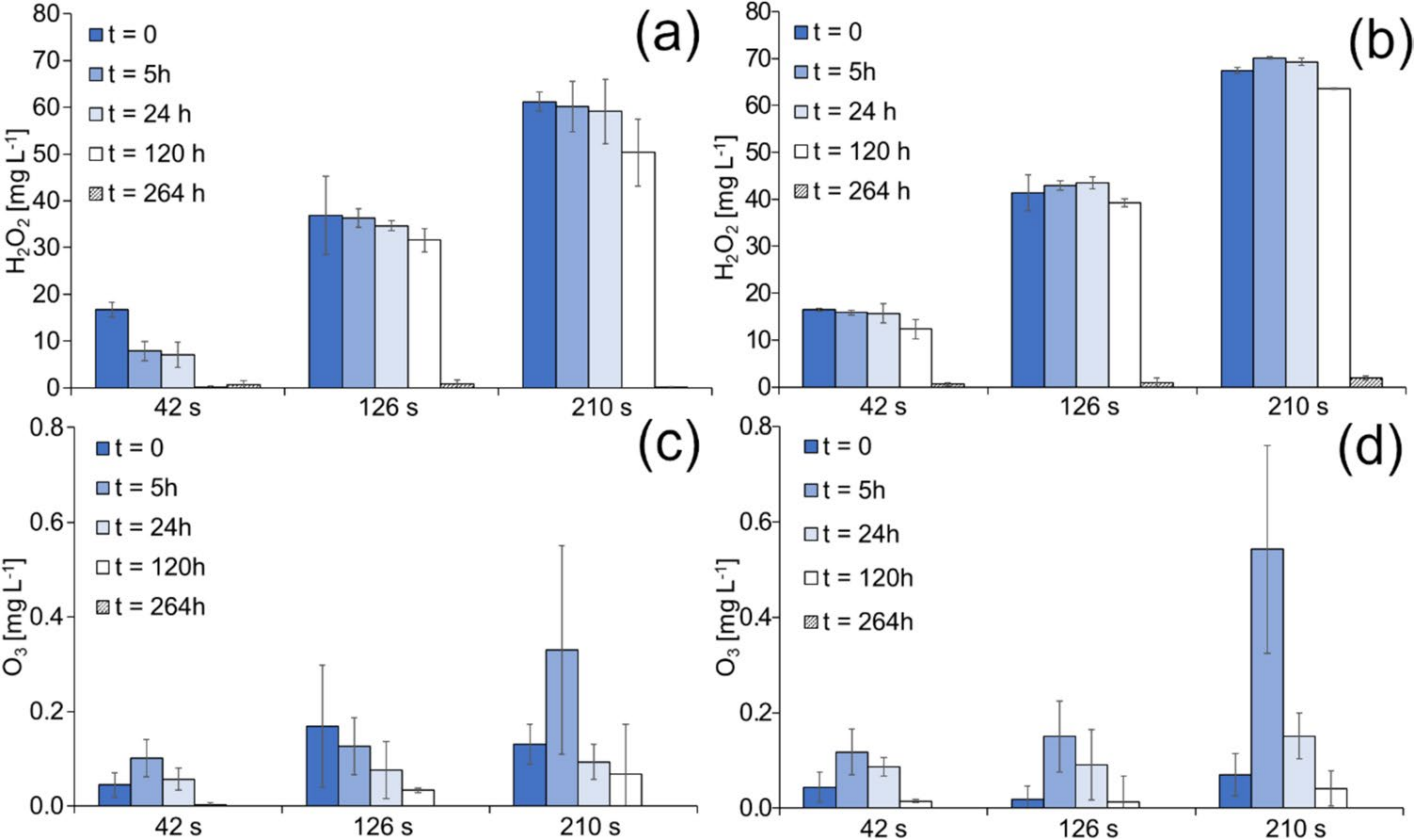
Effect of the presence of cyanobacteria (**a**, **c**) *Synechococcus elongatus* and (**b**, **d**) *Merismopedia minutissima* on the yield and changes of hydrogen peroxide and ozone content in media (treated after inoculation)

In the second experiment, the medium was treated after inoculation, showing the direct effect of CP on cyanobacterial cells. In this case, the medium was inoculated with cells to reach the same chlorophyll concentration (150 ± 25 mg L^−1^). In this case, we also tested whether the content of H_2_O_2_ and O_3_ is dependent on treatment time and how the density of these ROS was affected by the presence of microorganisms. Figure 6a and b indicates that the concentration of hydrogen peroxide is proportional to the duration of treatment. The presence of cyanobacteria strongly affects the stability of H_2_O_2_, resulting in a decrease as early as the fifth day after treatment and finally reaching zero after the eleventh day. It is unsurprising that H_2_O_2_ is gradually depleted in the presence of microorganisms and organic matter that results from their degradation.

Similar to the ZBB medium alone, the evolution of the ozone content showed maxima after 5 h (especially in the case of the 210-s treatment) and lasted at least 5 days in solution.

To sum up, the presented long-lived ROS, we have shown that the promising application potential of CaviPlasma technology is based on its high energy efficiency, i.e., G(H_2_O_2_) ≈ 10 g kWh^−1^ and G(O_3_) ≈ 0.03 g kWh^−1^ (in deionised water). Ozone production in CaviPlasma was even higher when compared to Gorbanev et al. (2016), who found that O_3_/^1^O_2_/O is only delivered into the liquid sample if O_2_ was present in the feed gas (e.g., only a negligible amount of these species can be formed from water molecules). Furthermore, this technology also stands out with impressive throughput rates of over 1 m^3^ per hour for a laboratory-scale treatment unit. Although bubble or aerosol-liquid plasma systems have similar energy efficiencies (Locke and Shih 2011; Wandell and Locke 2014), they have significantly lower throughputs (typically tens of millilitres per minute) compared to CaviPlasma technology (Anpilov et al. 2001; Wandell and Locke 2014).

### Nitrite content

Nitrite content was determined immediately after treatment in different matrices as an indirect indicator of nitric oxide radicals, as the production rate of gaseous nitrogen oxides strongly correlates with that of aqueous nitrites and nitrates (Hu et al. 2022; Machala et al. 2019). Although the method can detect trace amounts (0.005—0.1 mg L^−1^ NO_2_), no nitrite was detected in treated deionised water. The measured pH values of treated samples also validate these observations. The CaviPlasma discharge is generated in saturated water vapours inside cavitation cloud, where only a limited nitrogen concentration is present. This results in a slight shift of pH value (± 0.5 pH) contrary to the common acidification of PAW caused by the dissolved NO_x_ and their acids (pH values down to 2–3 are reported) (Verlackt et al. 2018). This fact is not only favourable from the application point of view, but it also contributes to the high temporal stability of the PTM in terms of H_2_O_2_ and O_3_ concentration. The pH value also plays a decisive role in determining the reactivity of the oxidants. Thus, plasma-treated water generated using CaviPlasma technology offers another advantage over the common plasma technologies producing plasma-activated water (Kaushik et al. 2019).

The presence of nitrite was confirmed in plasma-treated tap water and growth medium, with concentrations of 4 and 11 µg L^−1^ (after 210-s treatment). These solutions contained nitrate (6.9 and 22.9 mg L^−1^), and the conversion factor was approximately 0.5*10^−3^. Although, no nitrite was detected in deionised water enriched in nitrate (10 mg L^−1^). Therefore, we suggested that nitrite was most probably formed by reducing the already present nitrate in the presence of transition metals found in tap water and growth medium. Thus, the nitrite formed may contribute to the disinfecting effect of these PTM without significantly altering their pH.

### Cyanobacteria growth inhibition

**Fig. 7:**
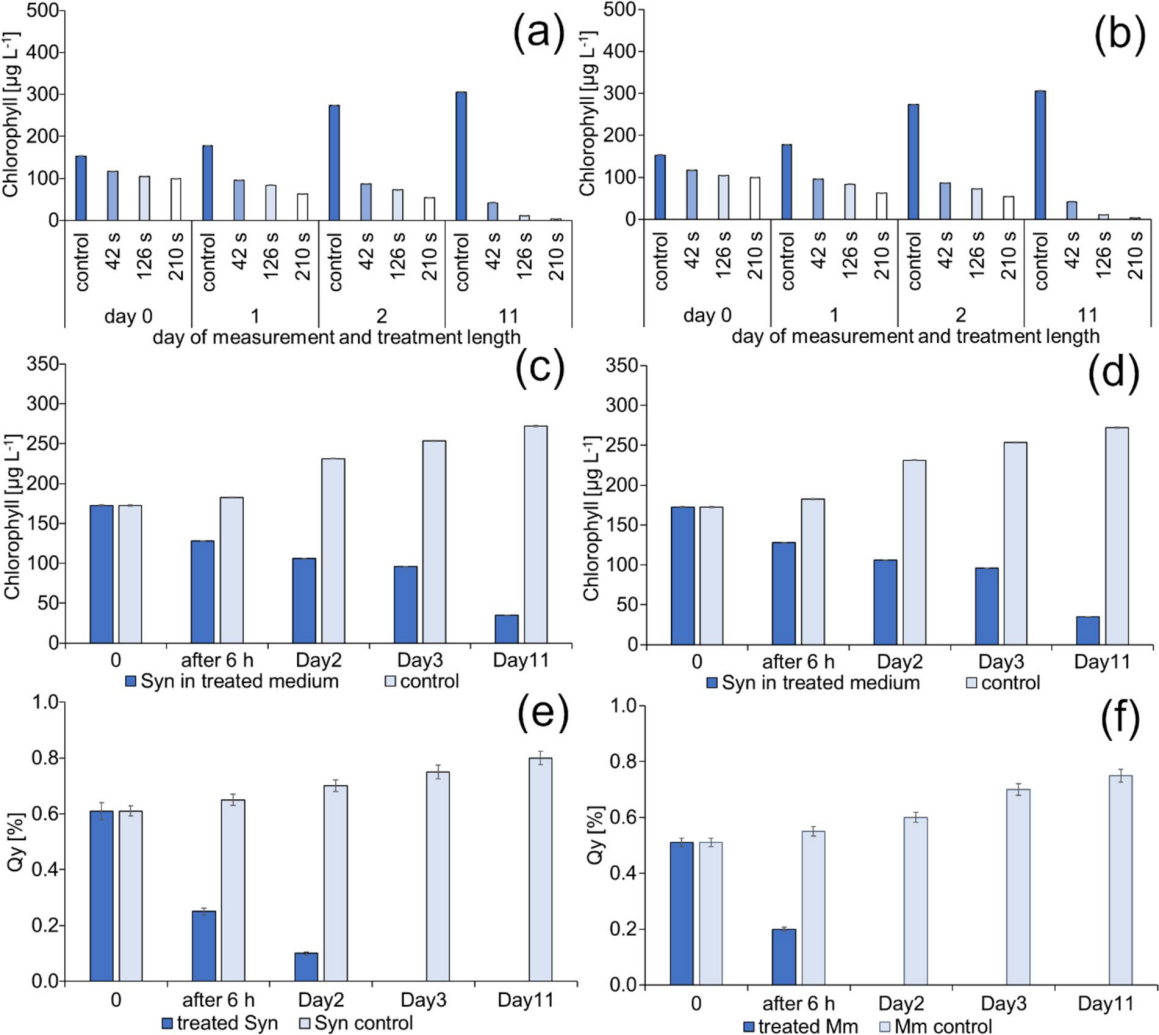
Changes of chlorophyll content in cell cultures of *Synechococcus elongatus* (**a**) and *Merismopedia minutissima* (**b**) after direct CP treatment. Changes of chlorophyll content in cell cultures of *Synechococcus elongatus* (**c**) and *Merismopedia minutissima* (**d**) after indirect CP treatment (210-s). Changes of photosynthetic activity expressed as a Quantum Yield (Qy) in cell cultures of *Synechococcus elongatus* (**e**) and *Merismopedia minutissima* (**f**) after indirect CP treatment

The results demonstrated in Fig. 7 suggest that oxidative stress induced by both direct and indirect (PTM) treatment can alter cyanobacteria's physiology and metabolic activities and affect their growth, reproduction, and survival. The efficacy of the treatment varies depending on the cyanobacterial species.

Figure 7 shows that photosynthetic vitality was reduced by 60% within six hours and reached zero value within three days at both cyanobacterial species.

Numerous studies indicate that both short and long-acting reactive oxygen/nitrogen species contribute significantly to the bactericidal effect (Di Mascio et al. 2019; Park et al. 2017; Zhao et al. 2020a, 2020b). Hydrogen peroxide is a widely accepted algicide in controlling cyanobacterial blooms. Because cyanobacteria lack catalases and ascorbate peroxidases, they are more susceptible to H_2_O_2_ than eukaryotic phytoplankton (Drábková et al. 2007; Weenink et al. 2015).

### Removal of microcystins

To explore CaviPlasma’s potential in MC removal, we studied the impact of three different treatment doses. To perform the study, we used deionised water enriched with microcystins (total 3.7 ± 0.3 ug L^−1^; concentration values of individual MC are shown in Table S2). The predominant MC in the water were MC-RR and MC-LR, representing 84% of total MC.

The formula for calculating the efficiency of microcystin removal (Eq. 2) and degradation kinetic curves (Eq. (3) of MC fitted by the pseudo-first-order reaction rate is as follows (Al Momani et al. 2008): 

2 $$\%\;of\;removal=(c_0-c_t)/c_o\times100\%$$

3 $$-\ln\;(c_t/c_0)=k\times t$$

where *c*_*0*_ and *c*_*t*_ are the initial and time concentrations of MC, respectively.

**Fig. 8:**
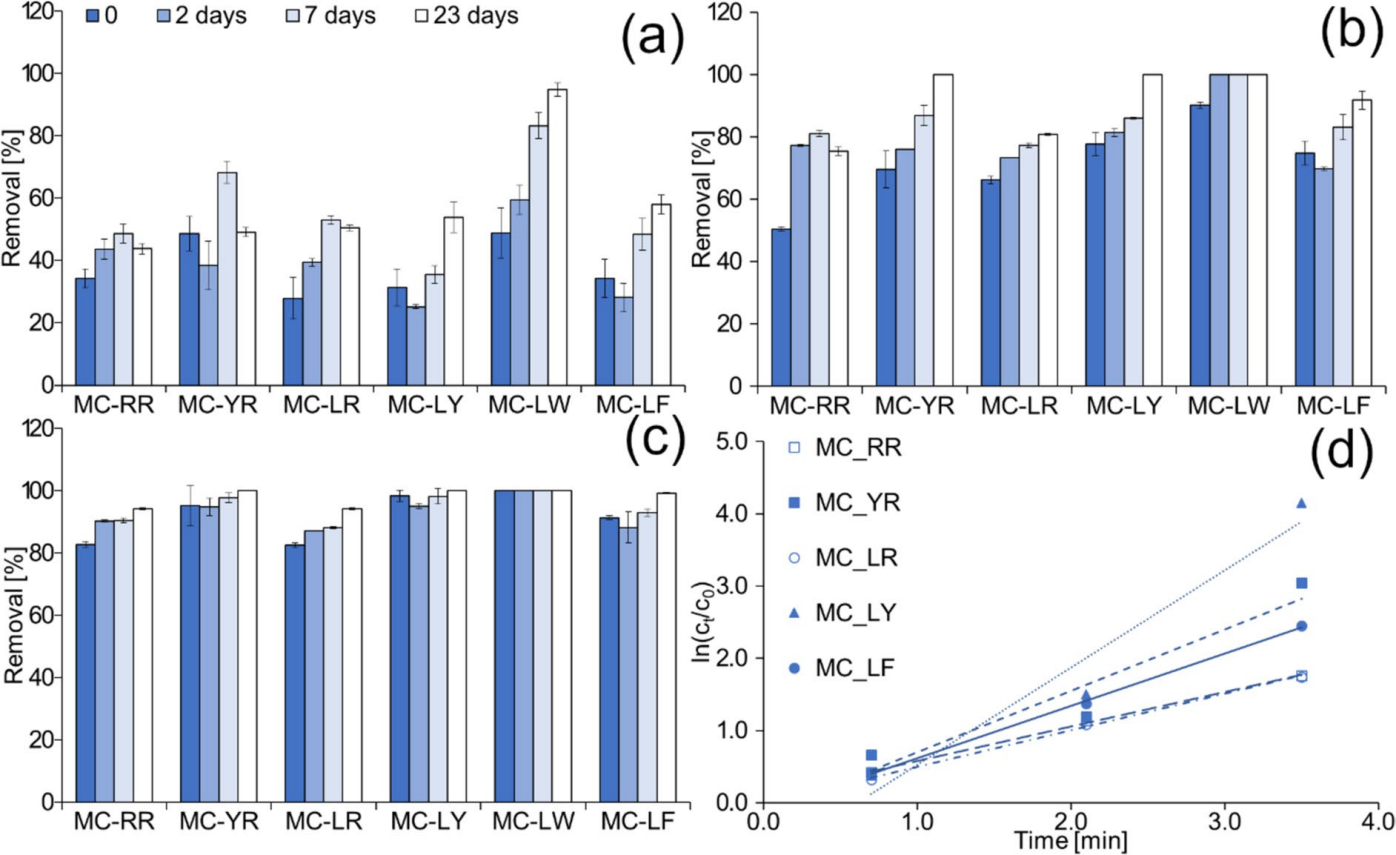
Microcystin removal after 42-s (**a**), 126-s (**b**), 210-s treatment (**c**), and their kinetic curves (**d**)

Similar to previous experiments, we observed the behaviour of hydrogen peroxide and ozone content and changes in the MC’s concentration over time (up to 23 days). The results of this experiment are presented in Fig. 8, which includes graphs for microcystin removal and their kinetic curves. The values of the MC's kinetic constants and correlation coefficients are given in Table 2. The H_2_O_2_ content trends after treatment are depicted in Fig. S4. The ozone was detected only after 210-s treated samples and reached a value of 0.29 mg L^−1^ and gradually decreased to 0.14 and 0.11 mg L^−1^ after two and seven days, respectively.

**Table 2:** The pseudo-first-rate constants of microcystins removal

Pseudo first-order kinetics	MC-RR	MC-YR	MC-LR	MC-LY	MC-LF
k [min^−1^]	0.59 ± 0.02	1.06 ± 0.34	0.63 ± 0.02	1.68 ± 0.39	0.91 ± 0.03
correlation coefficient	0.998	0.906	0.999	0.949	0.999

The data presented in Fig. 8a–c indicates that microcystin (MC) elimination increases as the treatment time increases. After 210 s of treatment, more than 84% of the total microcystin content is removed. In comparison, Wang et al. (2024) removed over 90% of microcystin-LR using dielectric barrier discharge after a treatment time of 40 min. Among the microcystins, the degradation order was MC-LY > MC-YR ≈ MC-LF > MC-RR ≈ MC-LR. The rate constants show that the oxidation process is structure-dependent, with MC that have an aromatic ring in the structure being preferentially degraded. This is typical of ozone-based oxidation, as reported by Sharma et al. (2012) or reaction with singlet oxygen (Schweitzer and Schmidt 2003). Wang et al. (2024) showed that e-aq, ·OH, H_2_O_2_, and O_3_ play a significant role in microcystin-LR degradation. They suggested that ·OH produced during plasma treatment attacks the conjugated diene and the aromatic ring on the Adda side chain, which undergo electrophilic substitution. Hydroxylation and by-products of diene-Adda double bond cleavage have also been detected by (He et al. 2015). These authors also confirmed that amino acid composition influences degradation kinetics and preferred reaction mechanisms. Additionally, the increasing conductivity in Fig. S4b supports the fact that the process of MC mineralisation is ongoing.

The post-treatment time also plays a role, as a significant decrease can be observed after seven days when the samples are kept under standard laboratory and light conditions in closed transparent glass bottles. In this case, even after a 42-s treatment, the percentage of total microcystin removal increased to 60%. Meanwhile, immediately after treatment, this value would be 31.5%. At the same time, the ozone content decreased by 25% in the case of the 126-s and 42% in the case of the 210-s treatment. In the case of the 42-s treatment, the ozone content was already below the quantification limit after 2 days of standing. As for peroxide, after seven days, the values decreased to 40%, 58% and 70% of the original values for the 42, 126, and 210-s treatments, respectively (Fig. S4a). The values of peroxide and ozone produced were one-third and one-half, respectively, compared to the deionised water in which the experiment was conducted.

## Conclusions

We have confirmed that the CaviPlasma device produces a wide range of reactive oxygen species in situ, mainly hydroxyl radicals and hydrogen peroxide. After the 210 s of plasma treatment (SIE 45 kJ L^−1^), H_2_O_2_ content was 84, 70 and 69 mg L^−1^ in deionised water, tap water and ZBB medium, respectively. Further, the CP device produces a considerable amount of O_3_, which correlates with approximately 270 times higher concentrations of hydrogen peroxide after 210-s treatment in deionised water. Singlet oxygen formation was also confirmed. The device offers several advantages, including high-volume throughput and no need for additional gas feeding. Moreover, there is no need to adjust the outlet’s pH after treatment, making the process environmentally friendly. CP technology thus presents substantial potential for real-world applications at industrial and commercial scales. It allows for smooth integration into existing manufacturing systems to enhance productivity and sustainability. Additionally, it can effectively address household water treatment needs, providing an accessible solution for improving water quality in everyday settings. Finally, we demonstrated on the cyanobacteria and cyanotoxins example that CaviPlasma technology is a highly efficient method which can destroy microorganisms and persistent toxins. In the case of cyanobacteria *Merismopedia minutissima*, zero photosynthetic vitality was observed 48 h after the 210 s CP modification. At the same treatment conditions, more than 84% of all total microcystins were removed immediately.

## Supplementary Information

Below is the link to the electronic supplementary material.

ESM1 (PDF 477 KB)

## Data Availability

Data will be made available on request.

## Footnotes

1 However, the matter in reaction chamber consisted of the liquid-voids mixture like a bubbly dispersed mixture, i.e., the hydrodynamic cavitation cloud (HCC) surrounded by the liquid phase. This results in the liquid fraction being substantially less than 100% and therefore the mass of the treated liquid had to pass through the reaction zone in a fact a considerably faster than the above stated upper estimation.
