# Supplementary material for: Exploring the dynamics of reactive oxygen species from CaviPlasma and their disinfection and degradation potential — the case of cyanobacteria and cyanotoxins
Source: Environ Sci Pollut Res Int. 2024 Dec 20;32(2):849–63. doi: 10.1007/s11356-024-35803-4 (PMC11732865; doi:10.1007/s11356-024-35803-4)
Supplement: Supplementary file 1 — (PDF 477 KB) [file 11356_2024_35803_MOESM1_ESM.pdf]

Supplementary information

Title: Exploring the Dynamics of Reactive Oxygen Species from CaviPlasma and their Disinfection and Degradation Potential – the case of cyanobacteria and cyanotoxins

Authors: Klára Odehnalová[a,*], Jan Čech[b], Eliška Maršálková[a], Pavel Sťahel[b], Barbora Mayer[b], Vinicius Tadeu Santana[c], Pavel Rudolf[d], Blahoslav Maršálek[a]

Affiliations:

[a]Department of Experimental Phycology and Ecotoxicology, Institute of Botany, Czech Academy of Sciences, Lidická 25/27, 602 00 Brno, Czech Republic

[b]Department of Plasma Physics and Technology, Faculty of Science, Masaryk University, Kotlářská 267/2, 611 37 Brno, Czech Republic

[c]Central European Institute of Technology Brno University of Technology Purkyňova123, 612 00 Brno, Czech Republic

[d]V. Kaplan Department, Faculty of Mechanical Engineering, Brno University of Technology, Technická 2896/2, 616 69 Brno, Czech Republic

Corresponding author:

Klára Odehnalová: klara.odehnalova@ibot.cas.cz; Institute of Botany, Czech Academy of Sciences, Lidická 25/27, 602 00 Brno, Czech Republic

Table of contents

- LC-MS/MS analysis of microcystins (page S3)
- Table S1 - Transitions of individual MCs (page S3)
- Procedure of solid phase extraction of microcystins (page S4)
- Figure S1 - Changes in the levels of hydrogen peroxide and ozone with the treatment time, and correlation of hydrogen peroxide and hydroxyl radical concentration (page S5)
- Figure S2 - The effect of sole-cavitation (Venturi) and synergistic action of discharge and cavitation (CaviPlasma) on hydrogen peroxide and ozone content (pageS6)
- Figure S3 - The effect of sole-cavitation (Venturi) and synergistic action of discharge and cavitation (CaviPlasma) on yield of hydroxyl radicals (page S6)
- Composition of ZBB medium (page S7)
- Table S2 - Concentration of individual microcystins in spiked water (page S8)
- Figure S4 – Changes of hydrogen peroxide concentration after treatment and conductivity with treatment time in deionised water spiked with MC (page S8)

LC-MS/MS analysis of microcystins

A Poroshell 120 EC-C18 column (2.1 x 100 mm; 2.7 μm) with a matching safety guard column (Agilent Technologies, CA, USA) was used for separation. Mobile phases A and B consisted of 0.1% formic acid in water and acetonitrile, respectively. The gradient elution was used for separation with increasing amounts of B fraction from 5 % to 55 % within 5 minutes and then to 90 % within 3 minutes, followed by a 4-minute hold. The flow rate was established at 200 μL min^{-1}, column temperature 45°C and the injection volume was set at 10 μL. The detector settings were configured as follows: capillary voltage at 4500 V, nozzle voltage at 500 V, gas temperature (N_2) at 350°C, gas flow at 10 mL min^{-1}, nebuliser at 20 psi, sheath gas temperature (N_2) at 350°C, and sheath gas flow at 12 mL min^{-1}. Mass data collection was performed using MassHunter Workstation software (Agilent Technologies, CA, USA) via multiple reaction monitoring (MRM) in positive mode. Transitions of individual MCs are listed in Table S1.

Table S1 Transitions of individual MCs

**Compound**	**CAS Number**	**Retention time (min)**	**Precursor ion (m/z)**	**Product ion[a] (m/z)**	**Fragmentor (V)**	**CE[b] (eV)**
MC-RR	111755-37-4	8.87	520	135/213	140	30/35
MC-YR	101064-48-6	9.39	523	135/70	90	50
MC-LR	101043-37-2	9.57	498	135/213	90	13/29
MC-WR	138234-58-9	9.68	535	135/103	100	13/69
MC-LA	96180-79-9	10.57	911	135/213	170	75/50
MC-LY	123304-10-9	10.60	1003	135/213	200	50/75
MC-LW	157622-02-1	10.97	1026	135/213	200	80/53
MC-LF	154037-70-4	11.18	987	135/213	170	78/68

[a] quantifier/qualifier, [b] collision energy

Solid phase extraction of microcystins

The SPE Oasis® HLB (500 mg, 6 mL) cartridges (Waters, Milford, MA) were conditioned using 5 mL of methanol followed by 5 mL of Milli-Q water. A portion of 50 mL of samples (pH = 5 - 6) was extracted under vacuum at a flow rate of approximately 5 mL min^{-1}. Afterwards, the sample holders were rinsed with 5 mL of distilled water and dried under vacuum suction for 20 min. Once extraction was completed, analytes were eluted with 5 mL of methanol and dried under a gentle nitrogen stream at 45°C. The dried extract was reconstituted in methanol/water (1:1, v/v) before LC-MS/MS analysis.

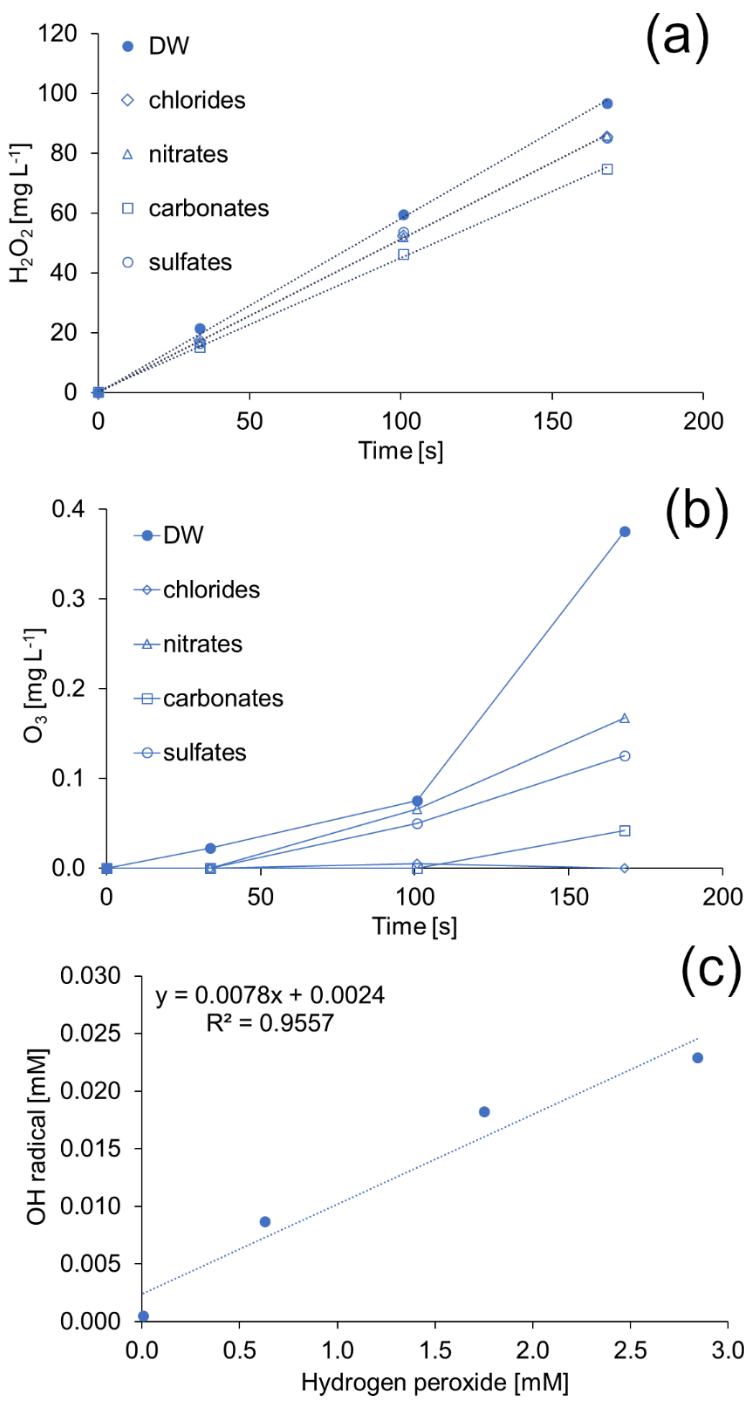

Fig. S1 Changes in the levels of hydrogen peroxide (a) and ozone (b) content as a function of treatment time in the presence of sodium salts. A four-litre batch was used in this experiment. Correlation of hydrogen peroxide and hydroxyl radical content in deionised water (c). Treatment times correspond to SIE of 8.4 kJ L^{-1}, 25.2 kJ L^{-1}, and 42 kJ L^{-1}

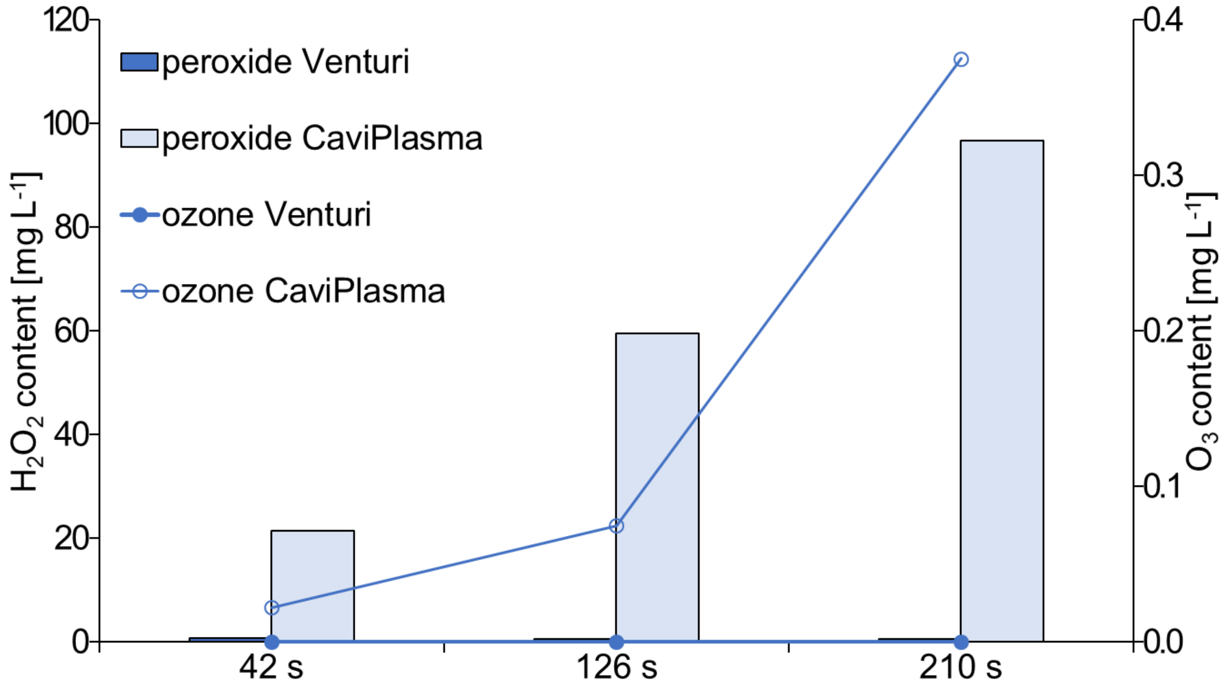

Fig. S2 The effect of sole-cavitation (Venturi) and synergistic action of discharge and cavitation (CaviPlasma) on hydrogen peroxide and ozone content

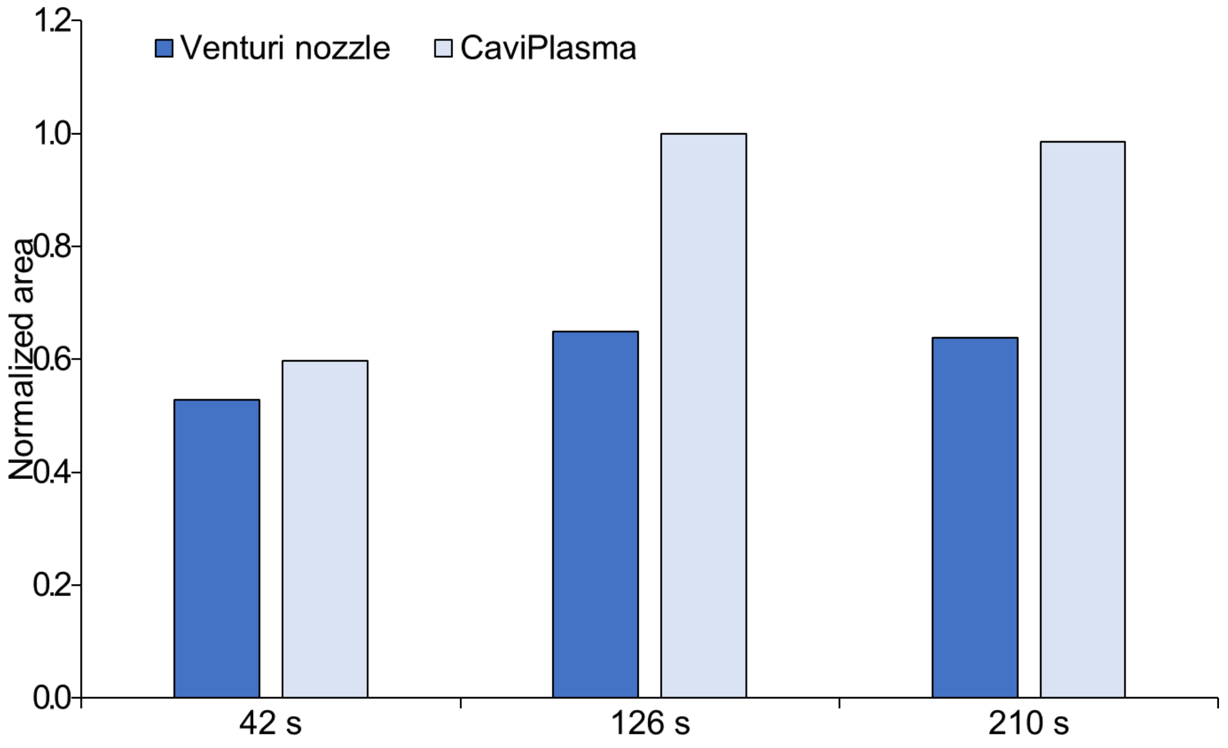

Fig. S3 The effect of sole-cavitation (Venturi) and synergistic action of discharge and cavitation (CaviPlasma) on yield of hydroxyl radicals

Composition of ZBB medium

Medium Z and BB were mixed in a 1:1 ratio (v/v) and diluted to 25%. The medium's conductivity and pH were 273.1 µS cm^{-1} and 7.0, respectively.

Medium Z	c [mg L^{-1}]
$NaNO_3$	467
$Ca(NO_3)_2 \cdot 6H_2O$	59
K_2HPO_4	31
$MgSO_4 \cdot 7H_2O$	25
Na_2CO_3	21
$NiSO_4(NH_4)_2SO_4 \cdot 6H_2O$	$1.6x10^{-2}$
$V_2O_4(SO_4)_3 \cdot 16H_2O$	$2.5x10^{-3}$
$(NH_4)_6 Mo_7O_{24} \cdot 4H_2O$	$7.0x10^{-3}$
$ZnSO_4 \cdot 7H_2O$	$2.3x10^{-2}$
$Cd(NO_3)_2 \cdot 4H_2O$	$1.2x10^{-2}$
$Al_2(SO_4)_3K_2SO_4 \cdot 24H_2O$	$3.8x10^{-2}$
$Na_2WO_4 \cdot 2H_2O$	$2.6x10^{-3}$
KBr	$9.5x10^{-3}$
H_3BO_3	$2.5x10^{-2}$
$MnSO_4 \cdot 4H_2O$	$1.8x10^{-1}$
$Cr(NO_3)_3 \cdot 7H_2O$	$1.2x10^{-2}$
KI	$6.6x10^{-3}$
$CuSO_4 \cdot 5H_2O$	$1.0x10^{-2}$
$FeCl_3$	2.8
Chelaton III	3.7
0.1N HCl	0.2 ml

Medium BB	c [mg L^{-1}]
$NaNO_3$	250
$CaCl_2 \cdot 2H_2O$	25
K_2HPO_4	75
KH_2PO_5	175
$MgSO_4 \cdot 7H_2O$	75
NaCl	25
Chelaton III	50
KOH	31
$FeSO_4 \cdot 7H_2O$	5.0
H_2SO_4 (conc.)	0.1
H_3BO_3	11.4
$MnCl_2 \cdot 4H_2O$	1.4
$CuSO_4 \cdot 5H_2O$	1.6
MoO_3	0.7
$ZnSO_4 \cdot 7H_2O$	8.8
$Co(NO_3)_2 \cdot 6H_2O$	0.5

Table S2 The concentration of individual microcystins in spiked water (determined by LC-MS/MS) for MC's removal experiment. The data expressed as average values ± standard deviation (n=4)

Fortified water	MC_RR	MC_YR	MC_LR	MC_LY	MC_LW	MC_LF
Concentration [µg L^{-1}]	0.67 ± 0.03	0.14 ± 0.02	2.46 ± 0.19	0.09 ± 0.01	0.16 ± 0.02	0.19 ± 0.01

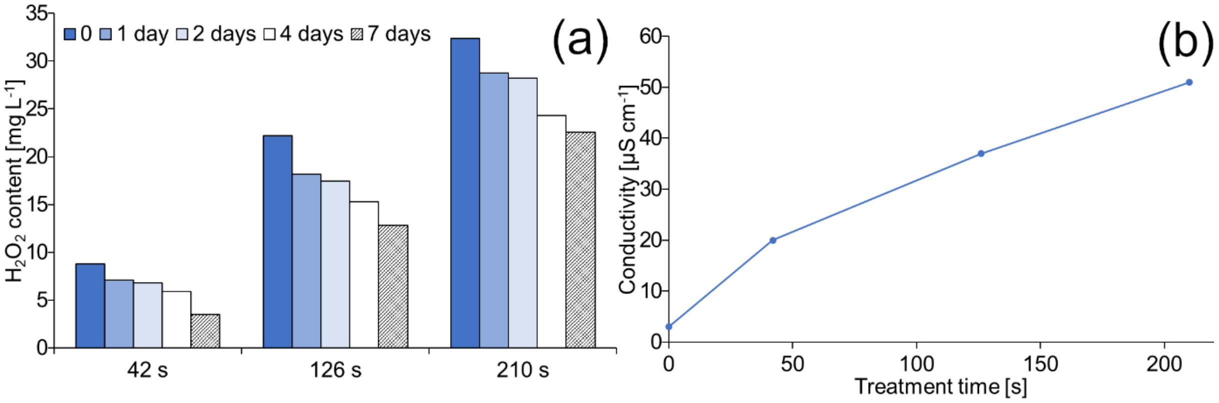

Fig. S4 Changes of hydrogen peroxide concentration (a) after treatment and conductivity with treatment time (b) in deionised water spiked with MC
